# Three-position model of segregations and critical grain sizes in nanocrystalline alloys

**DOI:** 10.3762/bjnano.17.105

**Published:** 2026-11-04

**Authors:** Ilya K Razumov

**Affiliations:** 1 N.M. Mikheev Institute of Metal Physics, Ural Branch of the Russian Academy of Sciences, Ekaterinburg, 620108, Russia

**Keywords:** critical grain sizes, grain-boundary segregation, nanocrystalline alloys, size effects, three-position model

## Abstract

A three-position thermodynamic model of grain-boundary segregations in binary and ternary alloys is proposed that accounts for the finite grain size and the energetic inequivalence of sites at the boundary. The classical McLean and Fowler isotherms are generalized to the case of three types of sites (i.e., with negative, near-zero, and positive segregation energy). The model parameters are matched to the first moments of the skew-normal spectra of segregation energies *E*_s_ obtained from atomistic and machine-learning calculations. For concentrated systems and ternary alloys, the equilibrium concentrations are obtained by minimization of the free energy of a system consisting of the bulk and grain boundaries, and the critical grain sizes are determined. The critical size *L*_cr_^(1)^ is the grain size at which the grain interior is practically purified, the bulk concentration falling to nearly zero, while the boundary concentration undergoes a sharp break; *L*_cr_^(2)^ is the grain size at which the integral degree of demixing is maximal. Both depend on temperature and on the average composition. In the dilute weak-segregation approximation, analytical expressions are also given for two characteristic sizes, *L*_c_^(1)^ (half-depletion radius) and *L*_c_^(2)^, which locate a smooth crossover in this case. It is shown that, in the presence of sites with a tendency to antisegregation, the average boundary concentration in the low-temperature limit tends to a value lower than unity; with decreasing grain size, the impurity is exhausted first in the bulk and then in the sites with segregation energies of smaller absolute value. In ternary systems with competition for the same sites, “locking” effects of the components appear and the concentration–grain-size dependences can become non-monotonic. Size-effect analysis is performed for Ni–Cu and Ni–(Pd, Pt) alloys. A generalized phase diagram of the nanocrystalline Ni–Cu alloy is constructed that demonstrates the suppression of decomposition and the stabilization of the solid solution upon grain-size reduction. The model reveals qualitative features of segregation size effects that are inaccessible within the single-site approximation and can serve as a basis for the analysis of thermodynamic stability of nanocrystalline alloys.

## Introduction

The service properties of alloys, that is, strength, ductility, creep resistance, corrosion resistance, and electrical characteristics, are largely determined by grain-boundary segregations of impurity and alloying elements [1–4]. Impurities such as P, S, Sn, and Sb cause intergranular embrittlement of iron, whereas C, B and certain transition metals enhance the cohesion of grain boundaries (GBs), thereby improving the strength of steels [5–6]. In addition, grain-boundary segregations (GBSs) promote grain refinement under external loading or stabilize the grain structure upon heating [7–8] and, in a number of alloys, control the equilibrium grain size that can be attained [9].

In the nanocrystalline state, when the fraction of atoms located near grain boundaries becomes comparable to the bulk fraction, the role of segregations increases qualitatively: They not only affect the local properties of the boundaries but also determine the thermodynamics of the alloy as a whole [10–15]. In [14] it was shown that, in an ideal or weakly non-ideal solid solution at moderate temperatures, critical grain sizes 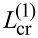 and 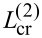 exist. For *L* < 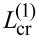 the grain interior is practically purified: The bulk concentration falls to nearly zero, and the boundary concentration undergoes a sharp break. When the condition *L* = 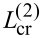 is fulfilled, the integral degree of demixing of the alloy reaches its maximum. In [15] an analysis was performed for a solid solution possessing a thermodynamic driving force for decomposition; a critical size 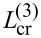 was identified at which the development of decomposition at the grain boundary is suppressed. In the same work, a generalized phase diagram of the alloy was constructed that depends on grain size and comprises several regions with different morphologies of GBSs and precipitates.

Classical models of grain-boundary segregation [16–17] treat the grain boundary as a homogeneous layer of material with modified thermodynamic properties. The change in the internal energy of the alloy upon transfer of an impurity atom from the bulk to the GB is called the segregation energy, *E*_segr_ = *E*_GB_ – *E*_bulk_. The McLean equation [16] is regarded as valid for an ideal solid solution (no interaction between impurity atoms) or for a strongly dilute solution (when the dependence of *E*_segr_ on impurity concentration may be neglected). The Fowler equation [17] takes into account the interaction of impurity atoms within the regular-solution model [18], including the change of the interaction parameter at the GB, and therefore describes segregation in non-ideal and/or concentrated solutions. These approaches were employed in [10–15].

However, already in [19–22] it was noted that the representation of a GB as a homogeneous layer often does not correspond to reality; in particular, a spectrum of solute binding energies was introduced in place of a single value [19–20]. A real grain boundary is a collection of atomic sites whose properties differ substantially both from the sites in the bulk and from one another. Some sites lie in regions of local compression, others in regions of local lattice expansion. Moreover, in the GB “core”, the coordination number and bond angles can vary considerably. Consequently, a correct description of GBS requires the introduction of a spectrum of segregation energies 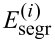 with weights corresponding to the occupation numbers of the inequivalent sites at the GB.

The importance of accounting for GB heterogeneity is particularly evident in the case of Co segregation at Cu grain boundaries. Emission Mössbauer spectroscopy indicates weak antisegregation of cobalt (segregation coefficient less than unity) [23], whereas the radiotracer method yields strong segregation, *E*_segr_ = −34.7 kJ/mol [24]. Combined diffusion of the isotopes ^57^Co and ^110m^Ag led to the conclusion that Co segregates not in the GB core but in the adjacent layer (so-called “sub-interface segregation”) [24]. This points to a relatively wide GB in which the segregation energy changes from weakly positive to negative with increasing distance from the interface plane. As a result, the effective value of *E*_segr_ becomes concentration-dependent not only because of impurity–impurity interactions but also because, at high impurity concentrations, all low-energy sites at the GB become occupied.

In [25], the segregation energies of various impurities (Ag, Au, Se, Ge, Ni, Co, Bi) at copper grain boundaries were calculated by density-functional theory (DFT) and compared with radiotracer data. Impurities of one group (Ag, Au, Bi) were found to occupy substitutional sites in the GB plane; impurities of a second group (Se, Ge) occupy sites immediately adjacent to the GB core; impurities of a third group (Ni, Co) exhibit weak antisegregation, which is somewhat at variance with the experimental “sub-interface segregation” [24] and may be related to the limitations of the DFT calculations (zero temperature, absence of anharmonic effects).

In [26], the concept of a spectrum of grain-boundary segregation energies in a polycrystal (taking into account the diversity of GB types) was introduced and shown to be well described by a skew-normal distribution. The methodological basis of the approach is atomistic modelling by molecular statics with empirical potentials (embedded atom method, EAM), which permits the calculation of segregation energies for a large number of individual atomic sites at the GB. In [27–28], machine-learning methods employing a limited set of high-accuracy data (including DFT) were used to generate segregation-energy spectra for a large number of binary systems. A typical situation is that both positive and negative values of 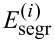 are present in the GB region.

In [29], the spectral approach was applied to materials with finite grain size. The authors demonstrated that, with decreasing grain size, the impurity preferentially occupies the low-energy sites (owing to the increase in their relative fraction) and that the contribution of triple junctions, which possess their own spectra, also grows. Consequently, the effective segregation energy that corresponds to the McLean model becomes grain-size dependent. A review of the spectral model of GBS is given in [30].

It is clear that the critical grain sizes examined in [14–15] (those corresponding to bulk purification, maximum degree of demixing, suppression of decomposition at the grain boundary, and attainment of the equilibrium grain size) change substantially in the presence of a dispersion of segregation energies, especially when 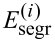 has opposite signs at different sites. The McLean and Fowler equations written with an effective segregation energy that depends on grain size in a non-obvious way become of limited informative value in this case. Numerical solution of a system of transcendental Fowler equations that employ a continuous spectrum of GB segregation energies is technically cumbersome and may yield physically opaque results. For physical analysis and approximate quantitative estimates, simple models that capture the essential features of the phenomenon and admit analytical or semi-analytical solutions are preferable.

In the present work, a simple generalization of the classical models that takes into account energetically inequivalent sites at the GB is proposed. The model considers three types of sites at the GB, that is, sites with negative, positive, and approximately zero segregation energy 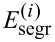. The values of 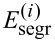 and the weight coefficients *w*_i_ that determine the relative fractions of sites of each type are chosen so as to match the continuous spectrum of segregation energies [27,29], ensuring agreement of the mean value, variance, and degree of asymmetry. Size effects in the development of grain-boundary segregations are investigated for the Ni–Cu and Ni–(Pd, Pt) alloys. Compared with the classical McLean–Fowler model, new qualitative conclusions are obtained: In binary alloys, with decreasing grain size, the impurity is first exhausted in the bulk and subsequently in the sites with a weak tendency to segregation at the GB, so that the GB properties depend on grain size in a non-trivial manner; in ternary alloys, non-monotonic and discontinuous behavior of the component concentrations at the GB as a function of grain size is possible, as well as “locking” of one of the components in the bulk. In addition, GB segregations promote the suppression of decomposition in the bulk, which is illustrated by a generalized phase diagram of nanocrystalline alloys.

## Results

### Three-site model of grain-boundary segregations in binary alloys taking into account grain size

We consider binary alloys in the absence of precipitation in the grain interiors. Let three types of energetically inequivalent sites be present at the grain boundary, namely, preferred for segregation (type 1), neutral or with a weak segregation tendency (type 2), and preferred for antisegregation (type 3). The free-energy density at the grain boundary *f*_GB_ is the additive sum of the contributions of these sites 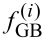. The free-energy densities in the bulk *f*_b_ and at each of the GB sites are written in the regular-solution approximation [18]:


[1]

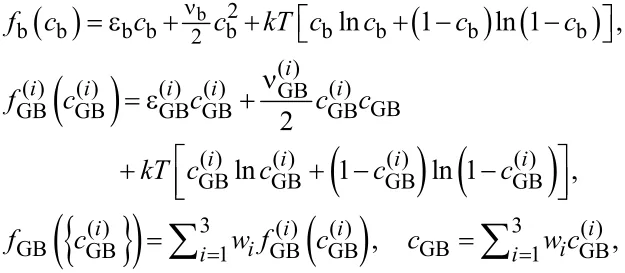



where 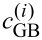 and *c*_b_ are the concentrations of the component at the *i*-th site on the GB and in the bulk, respectively, *c*_GB_ is the average concentration at the GB, 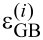, 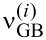 and ε_b_, ν_b_ are the coefficients of the expansion of the free energies in powers of the corresponding concentrations, and *w_i_* are the weight coefficients that determine the fractions of sites of the *i*-th type. The interaction of an impurity atom occupying an *i*-th site with its environment is treated in the mean-field approximation (contribution ~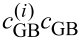); consequently, 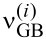 has the meaning of an effective interaction parameter for impurity atoms at sites of type *i*.

The boundary of the two-phase region (binodal) and the condition of loss of stability of the homogeneous state (spinodal) in the regular-solution model are described by the equations [18]:


[2] $$\begin{array}{l} \frac{1}{1-2c_{\text{eqv}}}\ln\left(\frac{1-c_{\text{eqv}}}{c_{\text{eqv}}}\right)=-\frac{\nu_{\text{b}}}{2kT},\\ 1+\frac{\nu_{\text{b}}}{kT}c_{\text{sp}}\left(1-c_{\text{sp}}\right)=0. \end{array}$$

Both curves *T*(*c*_eqv_) and *T*(*c*_sp_) have the shape of a dome with the vertex at *c*_eqv_ = 0.5, *kT* = −ν_b_/4. Above the binodal dome, the solid solution is stable; below the spinodal dome, it separates into two phases whose compositions are given by the roots of Equation 2 at a given *T*. Between the two curves lies the region of metastable states. In the present section, we restrict ourselves to the case in which the figurative point (temperature *T*, average composition *c*_0_) on the phase diagram of the alloy lies in the stability region (above the spinodal dome).

Under conditions of thermodynamic equilibrium the chemical potentials in the bulk and in all subsystems at the GB are equal:


[3] $$\mu_{\text{b}}=\mu_{\text{GB}}^{\left(i\right)},\;\mu_{\text{b}}=\frac{\partial f_{\text{b}}}{\partial c_{\text{b}}},\;\mu_{\text{GB}}^{\left(i\right)}=\frac{1}{w_{i}}\frac{\delta f_{\text{GB}}}{\delta c_{\text{GB}}^{\left(i\right)}},$$

where *w_i_* the fraction of atomic sites belonging to the *i*-th subsystem. When evaluating 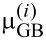, the variational derivative of the total free energy of the boundary is used because a change in the number of atoms in one site (owing to the interaction) affects the free energy of the remaining sites.

This yields the generalized system of Fowler equations [17] written for each of the sites at the GB:


[4] $$\frac{c_{\text{GB}}^{\left(i\right)}}{1-c_{\text{GB}}^{\left(i\right)}}=\frac{c_{\text{b}}}{1-c_{\text{b}}}\exp\left(-\frac{E_{\text{segr}}^{\left(i\right)}}{kT}\right),$$

[5] $$E_{\text{segr}}^{\left(i\right)}=E_{\text{s}}^{\left(i\right)}+\frac{1}{2}\nu_{\text{GB}}^{\left(i\right)}c_{\text{GB}}+\frac{1}{2}\sum_{k}w_{k}\nu_{\text{GB}}^{\left(k\right)}c_{\text{GB}}^{\left(k\right)}-\nu_{\text{b}}c_{\text{b}},$$

where 
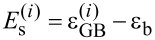
 is the segregation energy of the component at the *i*-th site (the difference of internal energies upon transfer of an impurity atom from the bulk to the GB) in the dilute solid solution, and ν_b_ and 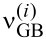 are the interaction parameters of impurity atoms in the bulk and at the GB, respectively. Note that, in the majority of works, instead of Equation 5, simpler (though less rigorous) approximations of the form 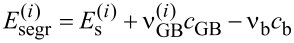 (coarse averaging over the sites) or 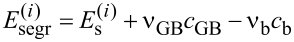 (the interaction energy at the GB is the same for all sites) are employed.

Thus, there are three equations (according to the number of site types) for four unknowns 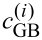 and *c*_b_. The closing condition is the law of conservation of matter:


[6] $$c_{\text{b}}\left(1-\phi \right)+c_{\text{GB}}\phi =c_{0},$$

where ϕ is the volume fraction of grain boundaries and *c*_0_ is the average impurity concentration in the grain. The quantities *w_i_* and 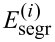 are external parameters of the model. They can be determined from calculated spectra of segregation energies [26–30] for specific alloys.

The integral degree of demixing of the alloy (0 ≤ *S*_d_ ≤ 1) is defined by the expression [14]:


[7] $$S_{d}=\frac{1}{2c_{0}\left(1-c_{0}\right)V}\int \left|c\left(r\right)-c_{0}\right|\text{d}V,$$

where *V* is the sample volume. For a homogeneous bulk and a homogeneous boundary (without 2D decomposition) this relation simplifies to


[8] $$S_{d}=\frac{\left(1-\phi \right)\left(c_{0}-c_{\text{b}}\right)}{c_{0}\left(1-c_{0}\right)}.$$

The total free energy of the system is the sum of the bulk and grain-boundary contributions:


[9]

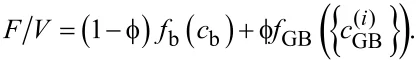



The volume fraction of grain boundaries for a spherical grain of radius *L* with a boundary layer of width *d* is given by


[10] $$\phi =1-{\left(1-\frac{d}{L}\right)}^{3}.$$

### Dilute solid solutions with weak segregations

In the limit of a dilute solid solution with weak segregations (*c*_b_ ≪ 1, 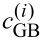 ≪ 1), the segregation energies depend only on the type of site at the GB, and the system of equations (Equation 4) is greatly simplified:


[11] $$\begin{array}{l} c_{\text{GB}}^{\left(i\right)}=c_{\text{b}}\beta_{i},\;c_{\text{GB}}=c_{\text{b}}\beta_{\text{eff}},\\ \beta_{i}=\exp\left(-\frac{E_{\text{segr}}^{\left(i\right)}}{kT}\right),\;\beta_{\text{eff}}=\sum_{i=1}^{3}w_{i}\beta_{i}. \end{array}$$

When 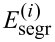 < 0, in the limit *T* → 0, one has β*_i_* → ∞, which restricts the range of applicability of the approximation not only to a sufficiently low initial impurity concentration but also to relatively high temperatures. From Equation 11, it follows that, within this approximation, the ratio of the occupations of sites of different types at the grain boundary 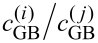 is determined solely by the difference of the segregation energies 
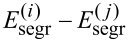
 and the temperature; it is independent of grain size. At the same time, the concentrations *c*_b_, *c*_GB_ themselves may depend on grain size. Indeed, from the conservation law (Equation 6) and Equation 11, one obtains the analytical expression for the bulk concentration:


[12] $$c_{\text{b}}=\frac{c_{0}}{1-\phi +\phi \beta_{\text{eff}}},$$

from which *c*_GB_ is then found according to Equation 10 and Equation 11.

In [26–30], it was proposed to approximate the spectra of segregation energies at the GB in a dilute solid solution by a skew-normal distribution:


[13] $$p\left(E_{\text{s}}\right)=\frac{1}{\sqrt{2\pi }\sigma }\exp\left(-\frac{{\left(E_{\text{s}}-\mu \right)}^{2}}{2\sigma^{2}}\right)\text{erfc}\left(-\frac{\alpha \left(E_{\text{s}}-\mu \right)}{\sqrt{2}\sigma }\right).$$

The parameters α, µ, and σ for specific systems are determined from spectra obtained on the basis of machine learning that employs first-principles calculations and molecular statics. Using the properties of the skew-normal distribution [31], one readily obtains an analytical expression for β_eff_(*T*):


[14] $$\beta_{\text{eff}}\left(T\right)=\int_{-\infty }^{\infty }\exp\left(-\frac{E_{\text{s}}}{kT}\right)p\left(E_{\text{s}}\right)\text{d}E_{\text{s}},$$

[15]

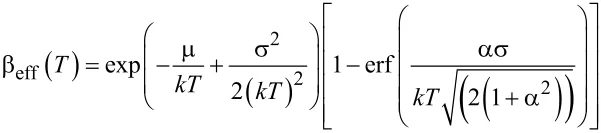



Substitution of Equation 10 and Equation 15 into Equation 11 and Equation 12 with the parameters α, µ, and σ taken from [26–30] makes it possible to examine the behavior of the concentrations *c*_b_ and *c*_GB_ for concrete alloys as functions of grain size and temperature.

From Equation 12 it follows that the dependence of the concentrations on grain size becomes appreciable when β_eff_ ≫ 1, that is, at sufficiently low temperatures. Equation 12 is a smooth function of the boundary fraction ϕ, and ϕ(*L*) itself varies smoothly, so there is no singular grain size in this approximation: *c*_b_(*L*) and *c*_GB_(*L*) remain convex and change gradually. What can be introduced is a characteristic radius 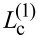 locating the crossover from the coarse-grain regime to the depleted regime. We define it by the condition 
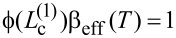
, which yields the expression:


[16] $$L_{\text{c}}^{\left(1\right)}\left(T\right)=d{\left[1-{\left(1-\frac{1}{\beta_{\text{eff}}(T)}\right)}^{1/3}\right]}^{-1}.$$

At this point, Equation 12 gives *c*_b_ = *c*_0_/(2 – ϕ) ≈ *c*_0_/2, while *c*_GB_ has fallen to about one half of its coarse-grain value *c*_0_β_eff_. Thus, 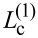 is the half-depletion radius, that is, the grain size at which the concentrations already differ by a factor of about two from the semi-infinite-grain limit. For *L* ≫ 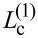 one has *c*_b_ ≈ *c*_0_ and *c*_GB_ ≈ *c*_0_β_eff_(*T*), practically independent of grain size; for *L* ≪ 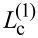 both concentrations continue to decrease, as *c*_b_ ≈ *c*_0_/(ϕβ_eff_) and *c*_GB_ ≈ *c*_0_/ϕ. These scales are qualitatively consistent with the estimates of [14]; the steepening associated with site saturation is absent here because the dilute weak-segregation approximation does not limit the capacity of the boundary.

For the integral degree of demixing *S*_d_(ϕ) we employ Equation 8 with the substitution of Equation 12. Differentiating the resulting dependence with respect to ϕ, we find the condition for a maximum of *S*_d_(ϕ):


[17] $$\phi_{\text{cr}}\left(T\right)={\left(1+\sqrt{\beta_{\text{eff}}(T)}\right)}^{-1}.$$

Hence, for a spherical grain and taking into account Equation 10, we obtain the characteristic size 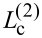 at which the degree of demixing is maximal:


[18] $$L_{\text{c}}^{\left(2\right)}\left(T\right)=d{\left[1-{\left(\frac{\sqrt{\beta_{\text{eff}}(T)}}{1+\sqrt{\beta_{\text{eff}}(T)}}\right)}^{1/3}\right]}^{-1}.$$

Thus, within the dilute-solution approximation, the characteristic sizes 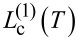 and 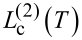 are independent of the impurity concentration. They should not be confused with the critical sizes mentioned in Section “Concentrated solid solutions with strong segregation driving forces”, which exist only beyond the present approximation and depend on composition.

To illustrate the model, we consider the segregation of Cu at Ni grain boundaries. The Ni–Cu alloy exhibits unlimited mutual solubility of the components above the dome of the two-phase region (*T* > 630 K) [32], which excludes competition between the development of GBS and processes of decomposition or intermetallic formation. The high solubility of copper persists at lower temperatures as well; for example, at *T* = 500 K it reaches 30 atom %. According to the data of [27], the energetic driving forces for segregation at the GB in this alloy are comparatively weak; therefore, up to room temperature the dilute-solution approximation with weak segregations remains adequate.

**Figure 1:**
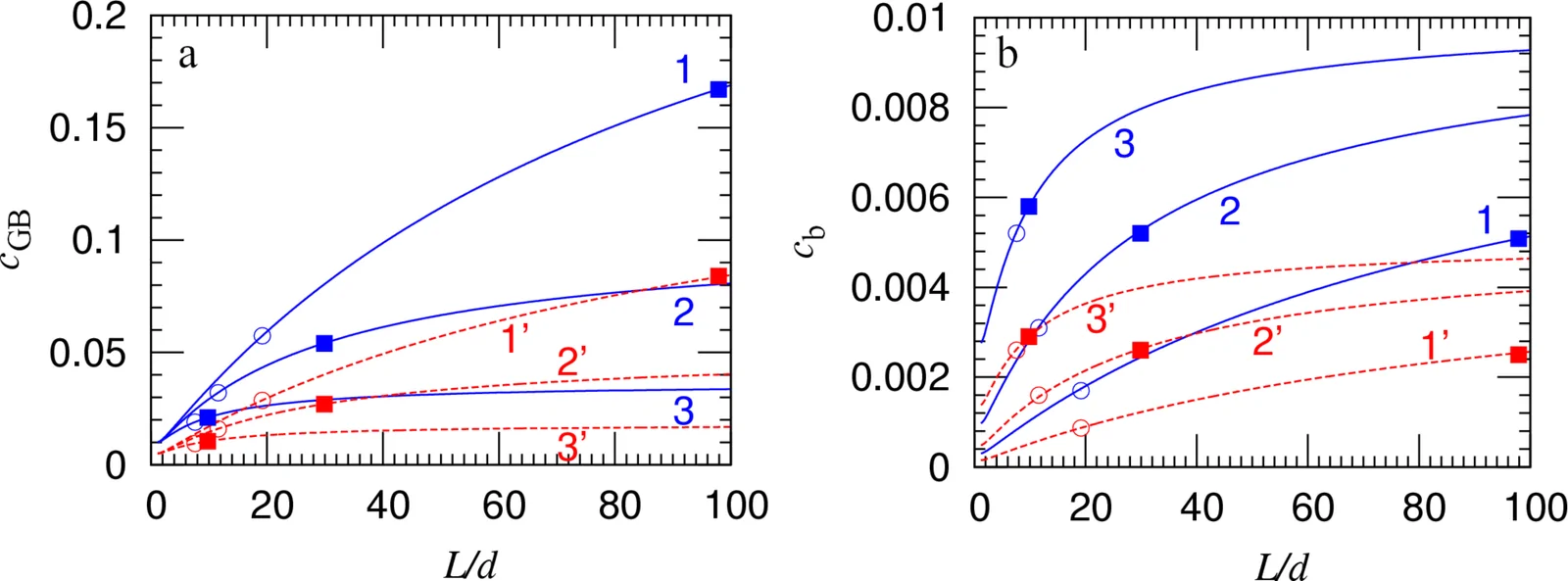
Dependences of the equilibrium concentrations of Cu at the boundaries (a) and in the interiors of Ni grains (b) on grain size at temperatures *T* = 400 K (1, 1′), 500 K (2, 2′), 700 K (3, 3′) and copper concentrations 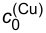 = 0.01 (1–3), 0.005 (1′–3′). The parameters of the segregation-energy spectrum are α = 0, µ = −2, σ = 8 kJ/mol [27]. Light and dark markers indicate the characteristic sizes 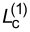 and 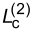, defined by Equation 16 and Equation 18 as the half-depletion radius and the grain size of maximum *S*_d_. They are reference points on smooth curves and do not mark a discontinuity.

Figure 1 shows the dependences of the equilibrium concentrations of Cu at the boundaries and in the interiors of Ni grains on grain size at various temperatures and average alloy compositions. The light and dark markers correspond to the characteristic sizes 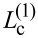 and 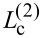, respectively. In coarse grains, the ratio *c*_GB_/*c*_b_ is of the order of 10 and is independent of grain size; on approaching and passing 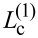, it decreases steadily with no discontinuity. Both characteristic sizes increase with decreasing temperature.

**Figure 2:**
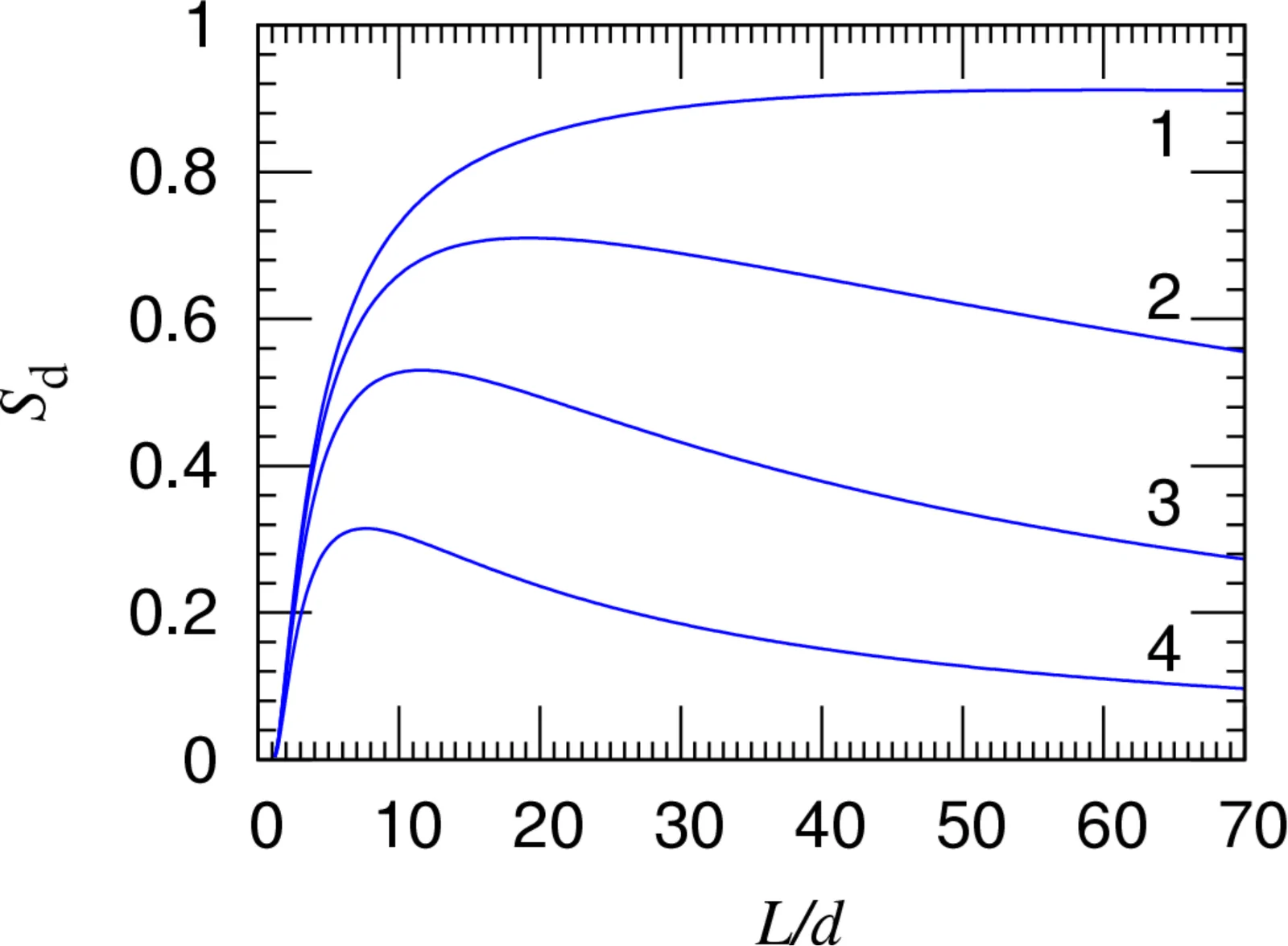
Dependence of the integral degree of demixing on grain size in the Ni–Cu alloy at *T* = 300 K (1), 400 K (2), 500 K (3), and 700 K (4).

**Figure 3:**
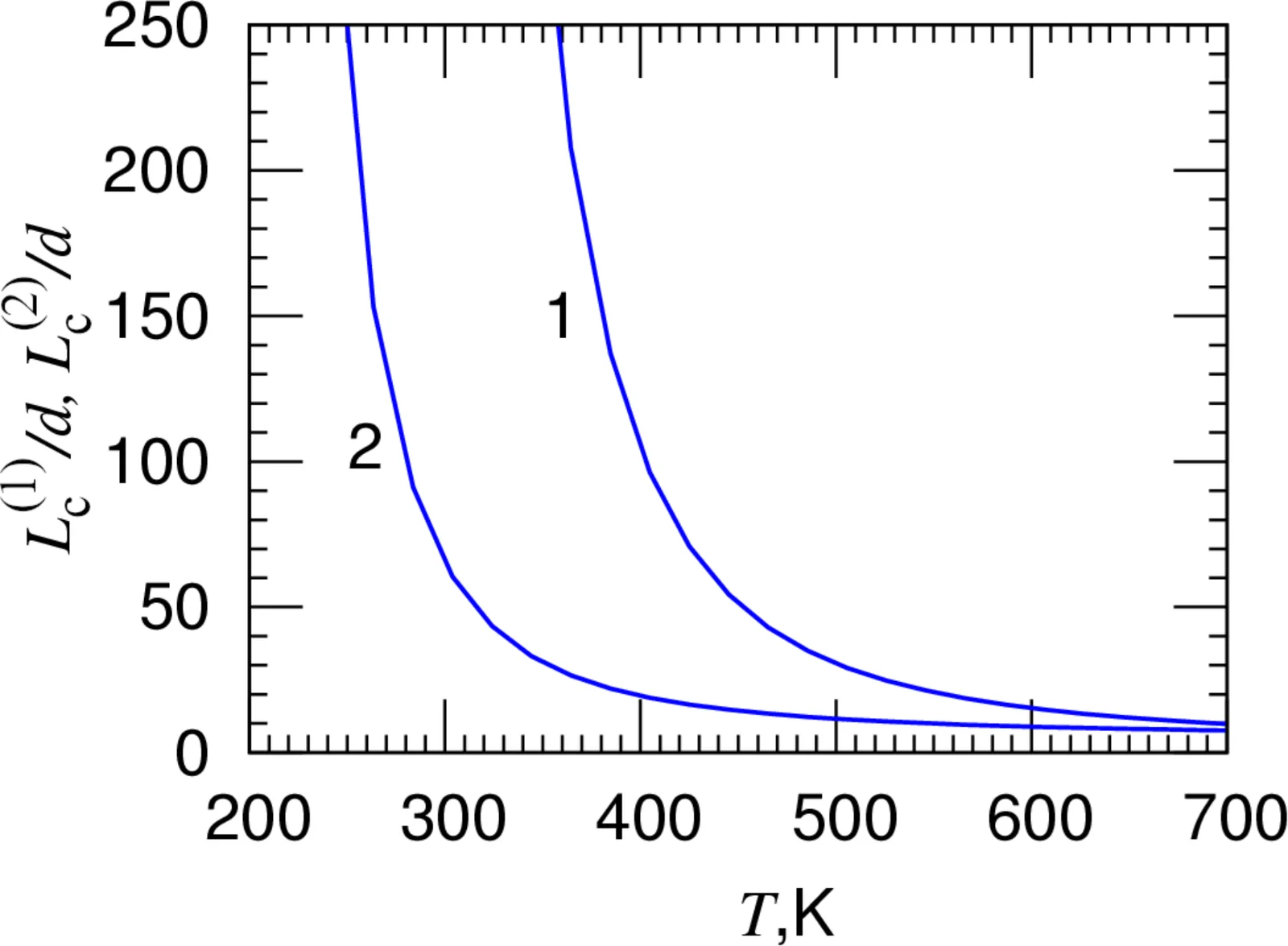
Temperature dependences of the characteristic sizes 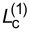 (curve 1) and 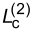 (curve 2) in the Ni-Cu alloy.

Figure 2 presents the dependences of the integral degree of demixing *S*_d_(*L*) on grain size at various temperatures, while Figure 3 shows the temperature dependences of 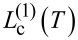 and 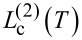 for the Ni–Cu alloy. Both sizes 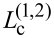 remain small (<50 nm) at *T* > 500 K, but rise sharply near room temperature. We emphasize that, in the low-temperature limit, the approximation employed becomes inapplicable because Equation 11 formally yields *c*_GB_ → ∞ as *T* → 0.

### Concentrated solid solutions with strong segregation driving forces

The classical McLean isotherm predicts that, for *E*_s_ < 0, the impurity concentration at the boundary tends to unity in the low-temperature limit. In contrast, in a model with several types of sites, after the sites with 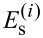 < 0 have been filled, saturation is reached, the sites with 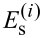 > 0 remain essentially unoccupied, and the average concentration at the GB can differ substantially from unity even as *T* → 0. This qualitative difference limits the possibility of introducing an “effective” segregation energy beyond the dilute-solution approximation with weak segregations (*c*_b_ ≪ 1, 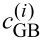 ≪ 1). The three-position model allows for a natural generalization of the spectral approach to the case of finite concentrations and strong segregation driving forces.

A continuous, normalized spectrum of segregation energies *p*(*E*_s_) can be approximately characterized by its first three moments (mean value *m*, variance *v*, and skewness γ):


[19] $$\begin{array}{l} m=\int_{-\infty }^{\infty }E_{\text{s}}p\left(E_{\text{s}}\right)\text{d}E_{\text{s}},\;v=\lambda_{2},\;\gamma =\frac{1}{v^{3/2}}\lambda_{3},\\ \lambda_{k}=\int_{-\infty }^{\infty }{\left(E_{\text{s}}-m\right)}^{k}p\left(E_{\text{s}}\right)\text{d}E_{\text{s}}. \end{array}$$

The corresponding expressions for the coarse-grained discrete model with three inequivalent sites are


[20] $$\begin{array}{l} m_{\text{d}}=\sum_{i=1}^{3}w_{i}E_{\text{s}}^{\left(i\right)},\;v_{\text{d}}=\lambda_{2}^{\left(\text{d}\right)},\;\gamma_{\text{d}}=\frac{1}{v_{\text{d}}^{3/2}}\lambda_{3}^{\left(\text{d}\right)},\\ \lambda_{k}^{\left(\text{d}\right)}=\sum_{i=1}^{3}w_{i}{\left(E_{\text{s}}^{\left(i\right)}-m_{\text{d}}\right)}^{k},\;\sum_{i=1}^{3}w_{i}=1, \end{array}$$

where *w_i_* are the weight coefficients of the inequivalent states.

In [26–30], the spectrum of GB segregation energies is expressed by a skew-normal distribution (see Equation 13), whose parameters are related to the moments *m*, *v*, and γ by the known relations [33]:


[21] $$\begin{array}{l} \delta =\frac{\alpha }{\sqrt{1+\alpha^{2}}},\;m=\mu +\sigma \delta \sqrt{\frac{2}{\pi }},\;v=\sigma^{2}\left(1-\frac{2\delta^{2}}{\pi }\right),\\ \gamma =\frac{\left(4-\pi \right)}{2}\frac{{\left(\delta \sqrt{2/\pi }\right)}^{3}}{{\left(1-\frac{2\delta^{2}}{\pi }\right)}^{3/2}}. \end{array}$$

The condition of optimal approximation of the spectrum by the discrete three-site model reduces to a system of four equations for the six unknowns 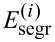 and *w_i_*:


[22] $$m_{\text{d}}=m,\;v_{\text{d}}=v,\;\gamma_{\text{d}}=\gamma ,\;\sum_{i=1}^{3}w_{i}=1.$$

We divide the sites at the GB into three groups with physically distinct properties, namely, those with a driving force for antisegregation (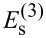 > 0), those with a weakly negative segregation energy (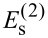 < 0), and those with a strong segregation driving force (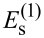 < 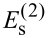). It is assumed that sites of the second type become substantially occupied by the impurity only at low temperatures and behave as “neutral” (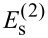 ≈ 0) at moderate temperatures.

The fraction of sites with positive segregation energy (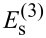 > 0) is determined directly from the spectrum:


[23]

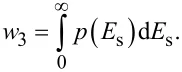



The integral in Equation 23 can be evaluated, for example, with the aid of the function CDF[SkewNormalDistribution[...],0] (Cumulative Distribution Function) in the Mathematica package.

The second missing condition for solving the system in Equation 22 is fixed by a physically reasonable approximation (the parameter σ characterizes the width of the spectrum; the factor 1/3 allows the negative part of the spectrum to be divided into two comparable intervals):


[24] $$E_{\text{s}}^{\left(2\right)}=-\sigma /3.$$

As a result of simple transformations, the quantities 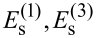 are expressed through the *w_i_* in explicit form from the equations *m*_d_ = *m* and *v*_d_ = *v*:


[25] $$E_{\text{s}}^{\left(1\right)}=\frac{m-w_{2}E_{\text{s}}^{\left(2\right)}-w_{3}E_{\text{s}}^{\left(3\right)}}{w_{1}},$$

[26] $$\begin{array}{c} E_{\text{s}}^{\left(3\right)}=m+\frac{w_{2}\left(m-E_{\text{s}}^{\left(2\right)}\right)}{w_{1}+w_{3}}\\ \pm \frac{1}{w_{1}+w_{3}}\sqrt{\frac{w_{1}}{w_{3}}\left[\left(w_{1}+w_{3}\right)v-w_{2}{\left(m-E_{\text{s}}^{\left(2\right)}\right)}^{2}\right]}. \end{array}$$

Finally, the equation γ_d_ = γ is solved numerically with respect to *w*_1_. Because of the coarsening of the spectrum, this solution sometimes proves to be physically inadmissible, leading to *w*_2_ < 0 or 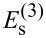 < 0. In such cases, we determine *w*_1_ not from the exact solution of the equation γ_d_ = γ, but by minimizing the residual *R*(*w*_1_) = (γ_d_ – γ)^2^ + (γ_2d_ – γ_2_)^2^, where γ_2_, γ_2d_ are the fourth moments (theoretical and of the discrete model) that allow the remote “tails” of the skew-normal distribution to be optimized:


[27] $$\gamma_{2}=2\left(\pi -3\right)\frac{{\left(\delta \sqrt{\left(2/\pi \right)}\right)}^{4}}{{\left(1-2\delta^{2}/\pi \right)}^{2}},\;\gamma_{2\text{d}}=\frac{1}{v_{\text{d}}^{2}}\lambda_{4}^{\left(\text{d}\right)}-3.$$

Table 1 lists the obtained values of 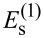 and *w_i_* for a series of Ni-based alloys; the parameters α, µ, and σ of the skew-normal distribution are taken from [27]. When the residual *R*(*w*_1_) is used, an asterisk (*) is placed next to the calculated value of *w*_1_.

**Table 1:** Parameters of the three-position model 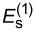 and *w_i_* for Ni-based alloys calculated from the parameters α, µ, and σ of the skew-normal distribution of segregation energies reported in [27]. When the residual *R*(*w*_1_) is employed, an asterisk (*) is placed next to the obtained value of *w_i_*.

Solute	α	µ, kJ/mol	σ, kJ/mol	*w*_1_	*w*_2_	*w*_3_	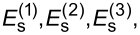 kJ/mol

Ag	−2.4	−13	56	0.60*	0.35	0.05	−83, −19, 46
Al	−0.4	−2	4	0.41	0.39	0.20	−7.5, −1.3, 2.1
Au	−1.7	−7	41	0.40	0.49	0.11	−71, −14, 0.07
Co	0.3	0	4	0.14*	0.27	0.59	−7, −1.3, 3.8
Cr	−3	2	16	0.29	0.55	0.16	−26, −5.3, 2.3
Cu	0	−2	8	0.38	0.22	0.40	−11, −2.7, 7
Fe	1.5	−5	11	0.10*	0.31	0.59	−16, −3.7, 8.5
Mg	−1.1	−41	62	0.67*	0.27	0.06	−111, −21, 44
Mo	−1.6	3	31	0.32	0.46	0.22	−49, −10, 11
Nb	0	−18	26	0.39	0.37	0.24	−48, −9, 17
Pb	0	−129	62	0.76*	0.22	0.02	−163, −21, 11
Pd	−1.6	10	25	0.25	0.38	0.37	−34, −8.3, 13
Pt	−1.7	−1	29	0.36	0.48	0.16	−48, −10, 6
Ta	−1.1	8	27	0.30	0.33	0.37	−37, −9, 17
Ti	−2.1	11	64	0.29	0.49	0.22	−100, −21, 20
V	−1.6	19	45	0.25	0.37	0.38	−60, −15, 24
W	−1.2	6	32	0.31	0.39	0.30	−48, −11, 17
Zr	−2.4	−9	47	0.59*	0.35	0.06	−68, −16, 37

**Figure 4:**
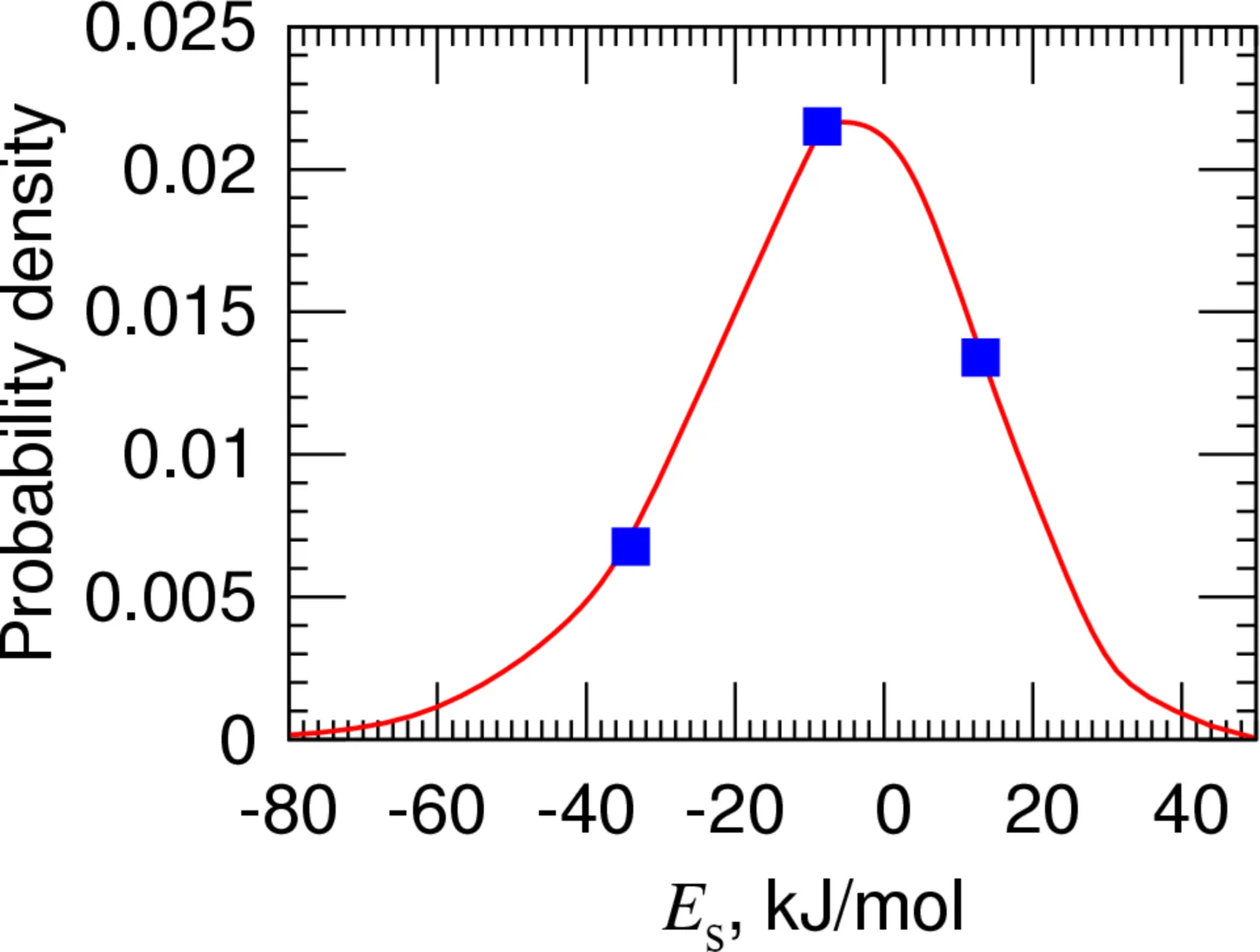
Spectrum of segregation energies of Pd at Ni grain boundaries according to the data of [27] (skew-normal distribution). The markers indicate the values of 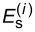 selected for the three-position model.

As an example, Figure 4 shows the skew-normal distribution of segregation energies of Pd at Ni grain boundaries calculated from Equation 13 with the parametrization of [27]. The markers indicate the values of 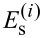 chosen for the three-position model.

In order to construct the dependences of the concentrations on grain size, *c*_b_(*L*), 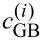
(*L*), and *c*_GB_(*L*), the obtained values of 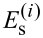 and *w_i_* must be substituted into the original system of Equations 4–6 and Equation 10.

A distinctive feature of a concentrated solution is the impossibility of neglecting the interaction energies of impurity atoms ν_b_ and 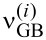. The bulk value ν_b_ can be obtained experimentally or from first principles, whereas the values 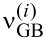 at different sites on the GB remain unknown in most cases. In [30,34–35], an atomistic approach was proposed for determining interaction energies directly at grain boundaries. However, this method remains resource-intensive and is still limited to individual systems (e.g., Al–Mg [34]). In the present work, we restrict ourselves to the approximation ν = ν_b_ ≈ 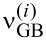, which allows us to rely on the known data on mixing energies. For example, in the Al–Mg alloy, according to [30], ν_b_ ≈ +56.6 kJ/mol (repulsion), while the effective Mg–Mg interaction energy at the GB is +54 kJ/mol.

As an example, we consider the Ni–Pd alloy, which possesses a sufficiently wide spectrum of segregation energies (see Table 1 and Figure 4) with pronounced driving forces both for segregation and for antisegregation at different sites in the GB. From the results of DFT calculations of mixing enthalpies [36], it follows that ν_b_ ≈ −20 kJ/mol. With this value of the interaction parameter, Equation 2 implies that the alloy forms a continuous series of solid solutions at *T* > 600 K, while a two-phase region exists below this temperature. Experimentally, the two-phase region in the Ni–Pd phase diagram has not yet been observed; this may be related to the low diffusion rate, the role of vibrational entropy (in addition to configurational entropy), and a possible overestimation of ν_b_ in the DFT calculations. Nevertheless, the present example is convenient for a qualitative analysis of size effects of segregation.

When the system of Fowler Equation 4 is solved numerically, the appearance of additional branches corresponding to unstable states cannot be excluded. Therefore the equilibrium concentrations were determined by minimization of the total free energy (Equation 9) with respect to the variables {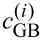} at fixed grain size *L*, subject to the conservation law (Equation 6) as an additional constraint.

**Figure 5:**
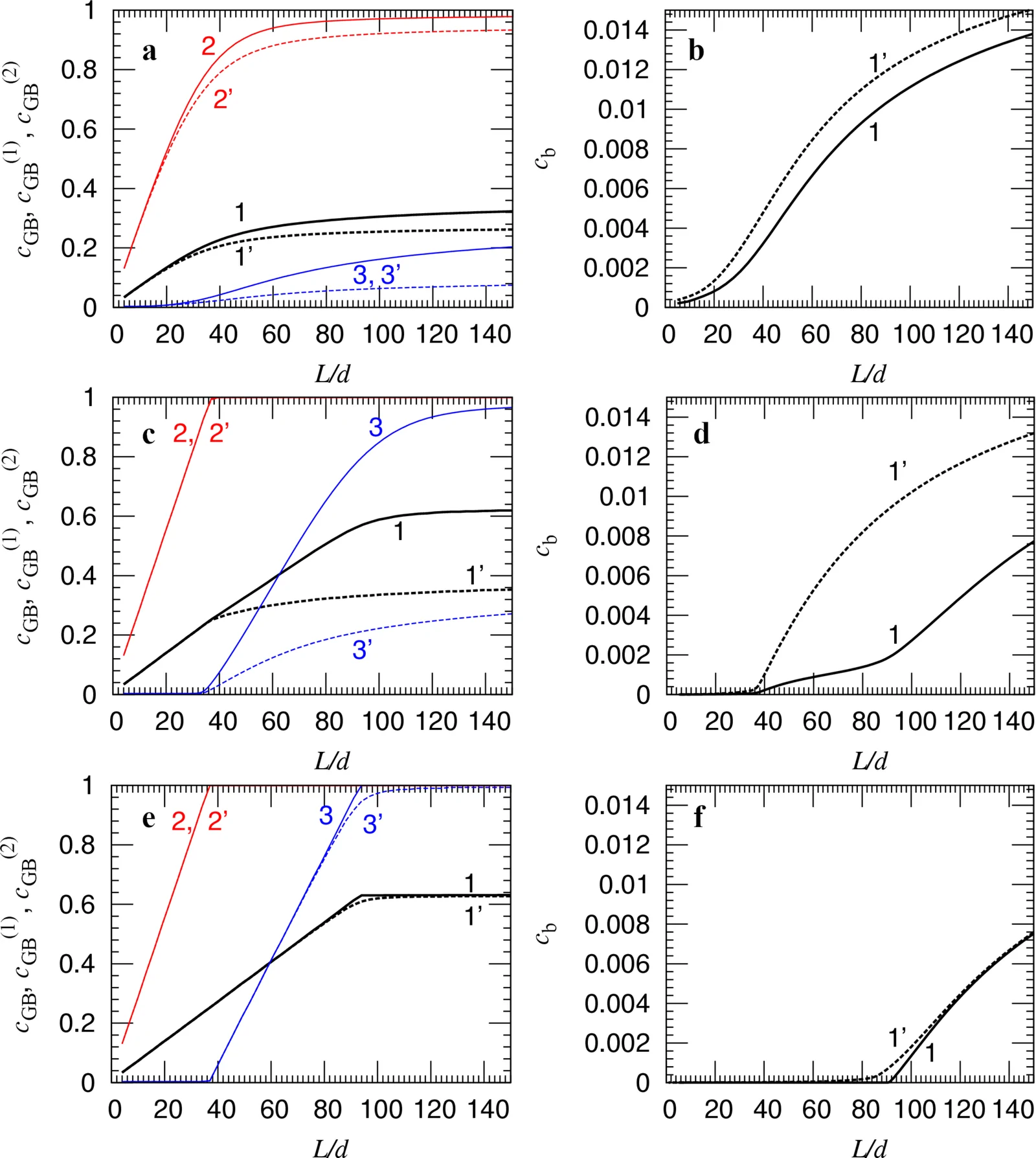
(a, c, e) Average equilibrium concentration of Pd at Ni grain boundaries *c*_GB_ (curves 1), concentrations at the first 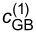 (curves 2) and second 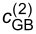 (curves 3) sites at the grain boundary as functions of grain size; (b, d, f) equilibrium bulk concentration *c*_b_ (curve 1) as a function of grain size; 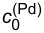 = 0.02; ν_b_ = 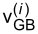 = −20 kJ/mol; *T* = 600 K (a,b), 300 K (c, d), 100 K (e, f). Curves 1′–3′ are constructed under the assumption of the absence of Pd–Pd interaction (ν = 0). In panels (e, f), the critical size is the grain size at which the boundary concentration breaks and the bulk concentration falls practically to zero.

Figure 5 shows the calculated average concentration 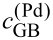 at the GB (Figure 5a,c,e, curves 1), the concentrations at sites of the first and second types (curves 2 and 3), and the bulk concentration 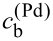 as functions of grain size for the average composition 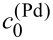 = 0.02. For comparison, the results for an ideal solution (ν_b_ = 0) are shown by dashed lines. The concentration of Pd at sites of the third type (with a tendency to antisegregation) is negligibly small in all cases and is not presented.

The obtained dependences reveal a number of qualitative features that are absent in the classical McLean–Fowler model: (1) At low temperatures, the average concentration of Pd at the grain boundary tends not to unity but to the value *c*_GB_ = *w*_2_ + *w*_3_ ≈ 0.63 (see Figure 5a,c,e, curves 1), because the sites of the third type (with a tendency to antisegregation) remain unoccupied in the limit *T* → 0. (2) Even in an ideal solid solution (ν_b_ = 0) at moderate and low temperatures, there exists a critical grain size 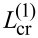 at which the impurity is exhausted in the bulk (*c*_b_ ≈ 0) (see Figure 5d,f, curves 1); upon further decrease of *L*, the value of *c*_GB_ falls rapidly (see Figure 5c,e, curves 1), in agreement with the conclusions of [14]. This behavior is qualitatively different from the weak-segregation approximation in the Section “Dilute solid solutions with weak segregations”, where the curves *c*_b_(*L*) and *c*_GB_(*L*) change monotonically with grain size, and the characteristic grain size 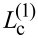 is not linked to any singularity on the curves. (3) The concentration at sites of the second type, 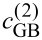, depends strongly on both temperature and grain size. At high *T*, it is negligible compared with 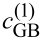 (Figure 5a, curve 3); at low *T* in coarse grains, it is close to unity, but falls rapidly starting from a certain grain size (Figure 5c,e, curve 3). At low *T*, as the grain size decreases, the impurity is first exhausted in the bulk and then in the sites of the second type at the GB, after which the concentration in the sites of the first type begins to decrease (see Figure 5e,f). The role of the Pd–Pd interaction is most significant at intermediate temperatures (Figure 5c,d). In the limit *T* → 0, the curves calculated with and without taking ν into account coincide: The magnitude of the segregations is determined solely by the average composition and the number of available sites.

**Figure 6:**
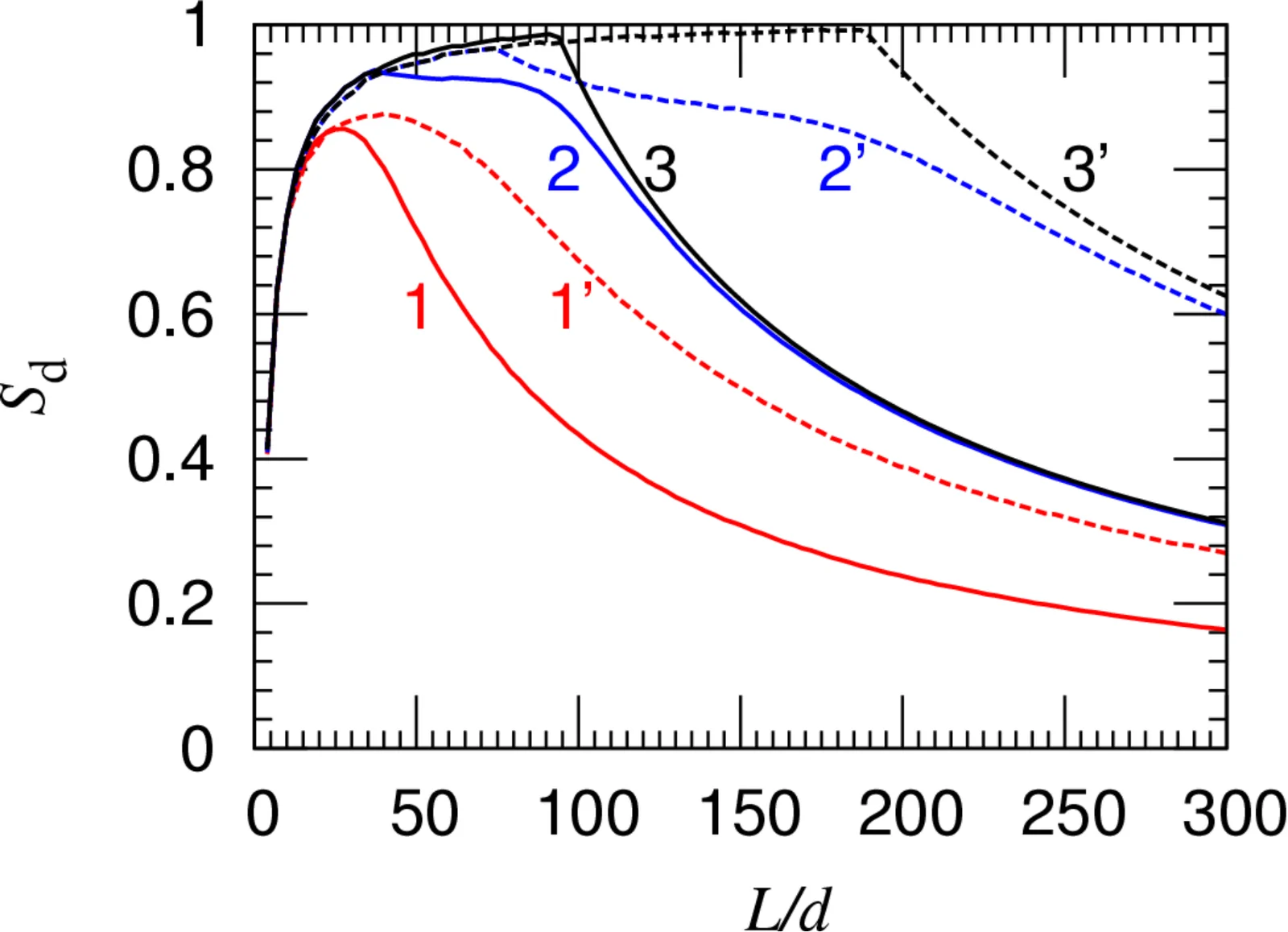
Integral degree of demixing of the Ni–Pd alloy as a function of grain size at *T* = 600 K (1,1′), 300 K (2,2′), 100 K (3,3′) at 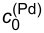 = 0.02 (1–3), 0.01 (1′–3′).

Figure 6 presents the dependence of the integral degree of demixing (Equation 8) on grain size. It can be seen that a decrease of the average composition 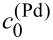 shifts the maximum of the curve toward larger grain sizes.

**Figure 7:**
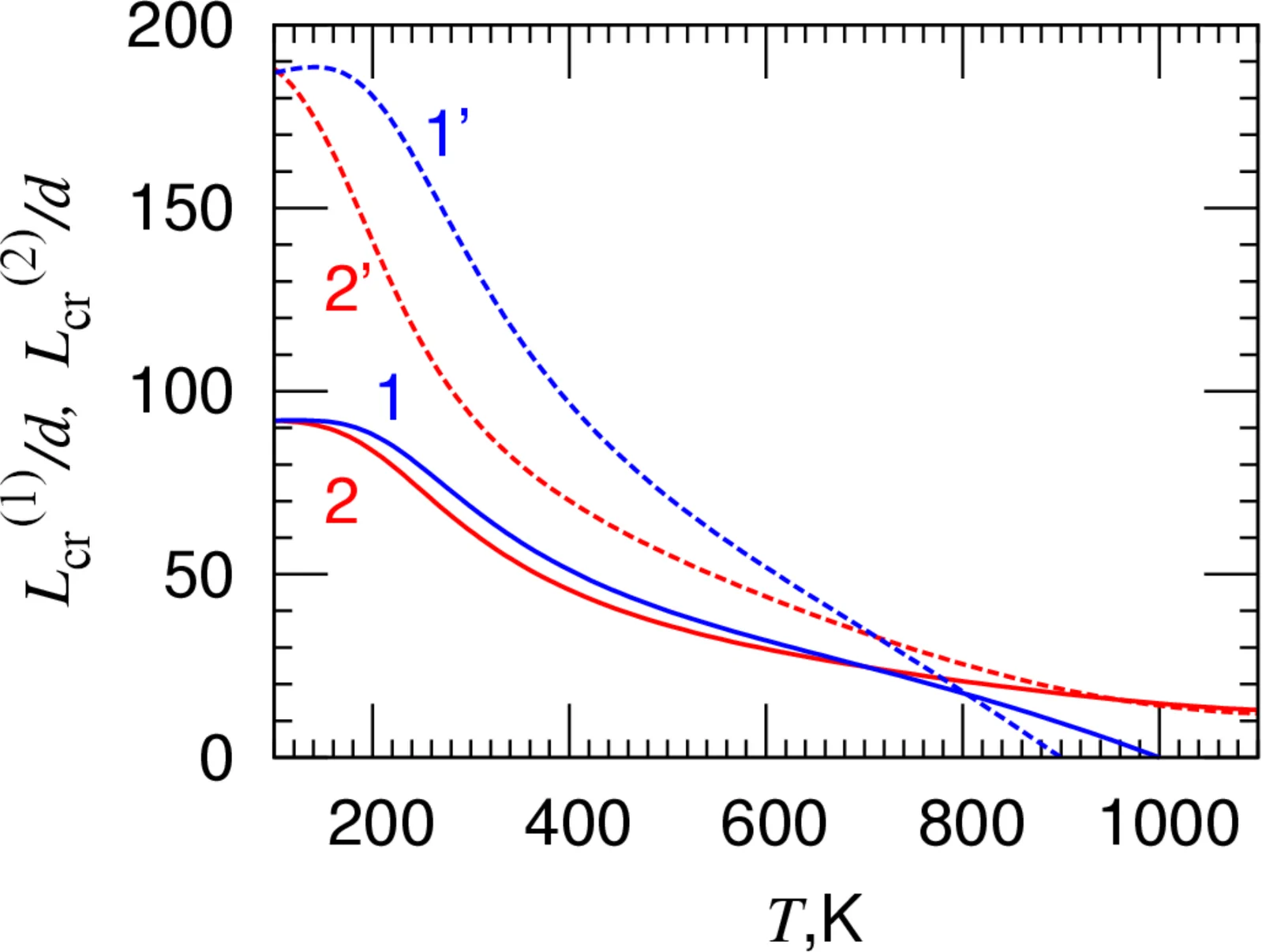
Critical grain sizes 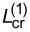 (curves 1, 1′) and 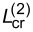 (curves 2, 2′) as functions of temperature in the Ni–Pd alloy for 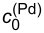 = 0.02 (1, 2), 0.01 (1′, 2′).

Figure 7 shows the temperature dependences of the critical sizes 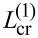 and 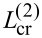 introduced in analogy to [14]. The size 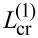 is defined by the inflection point of the curve *c*_b_(*L*) (when two inflection points are present, the one corresponding to the smaller *L* is chosen) and characterizes the condition of purification of the grain interior from the impurity; it exists only at sufficiently low temperatures. The size 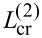 corresponds to the maximum of *S*_d_(*L*). In the low-temperature limit, 
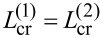
; at high temperatures, 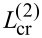 remains finite, while 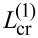 ceases to exist.

The significant qualitative distinction of the critical sizes 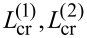 from the characteristic sizes 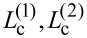 introduced in Section “Dilute solid solutions with weak segregations” (within the dilute-solution approximation with weak segregations) consists in their clear dependence on the average alloy composition (the smaller *c*_0_, the larger the critical sizes 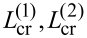) (compare curves 1, 2 and 1′, 2′ in Figure 7). Thus the three-site model, matched to the first moments of the skew-normal spectra of segregation energies, makes it possible to describe a number of qualitatively new size effects in concentrated solid solutions: (1) saturation of the average boundary concentration at *T* → 0 by the value *w*_1_ + *w*_2_ < 1; (2) successive exhaustion of the impurity first in the bulk and then in the sites of weak segregation energy; (3) dependence of the critical grain sizes on the average alloy composition; (4) substantial influence of the interaction parameter ν in the intermediate temperature range. These features are inaccessible within the classical McLean–Fowler model and must be taken into account when analyzing the thermodynamic stability of nanocrystalline alloys.

### Grain-boundary segregations in ternary alloys

The principal difficulty in generalizing the three-site model to multicomponent alloys is that atoms of different species may occupy either distinct or identical sites at the grain boundaries. This means that competition for the same types of sites arises between certain impurity atoms, while for other species such competition is absent. In other words, sites of low segregation energy for one component may be sites of high segregation energy for another component; the spectra of segregation energies of different components are, as a rule, non-isomorphic.

The most general and consistent approach requires that the sites at the GB be classified not according to the magnitude of the segregation energy of a given component, but according to their lattice equivalence. First, a complete list of inequivalent lattice positions at the GB must be compiled and their weight coefficients determined. Then, the segregation energies of each component at these sites must be calculated. Finally, a system of equations of the type of Equation 4 must be solved for the concentrations of all components in all sites. This full model can be substantially simplified in a number of practically important special cases: (1) A strongly dilute solid solution with weak segregations of components A and B: When *c*_b_ ≪ 1, 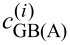 ≪ 1, and 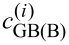 ≪ 1, competition (or cooperation) of impurities of different species at the GB may be neglected. Then, the average concentrations of each component at the GB and in the bulk are calculated independently from Equation 11 and Equation 12 using the corresponding spectra of segregation energies. (2) Strong segregations in the absence of competition for sites: Components A and B occupy different types of sites at the GB (sites with a driving force for antisegregation for one component are sites with a driving force for segregation for the other component). This situation is typical when the atomic radii of A and B differ strongly, especially if one of them is substantially larger and the other substantially smaller than the radius of the matrix atom. Large atoms tend to occupy sites with excess volume, small atoms occupy sites of local compression, which promotes relaxation of lattice strains. Such a situation is realized, for example, in the ternary alloy Ni–(Pd,Be). Pd atoms (larger than Ni) segregate to expansion sites, Be atoms (smaller than Ni) to compression sites. If the solute components A and B do not interact (ν_AB_ = 0), segregation of each of them at the grain boundary can be treated independently, that is, as in the corresponding binary alloy, using the parameters 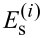 and *w_i_* obtained within the three-position model (Section “Concentrated solid solutions with strong segregation driving forces”). In the more general case, components A and B may interact both in the bulk and at the grain boundary [37], even in the absence of competition for sites. Accounting for this interaction does not change the set of weights 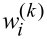, *k* = {A, B}, but shifts the segregation spectra, 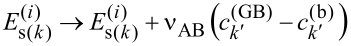, where 
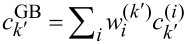
 is the average concentration of component *k*´ ≠ *k* at the grain boundary, evaluated with its own weights 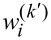. This shift coincides with Guttmann’s interaction coefficient [37] and does not assume shared segregation sites. For ν_AB_ < 0, the segregations reinforce each other, whereas for ν_AB_ > 0 they suppress each other. Since the shift for component A depends on the concentrations of B and vice versa, equilibrium must be determined by jointly minimizing the free energy of the sample, with the grain-boundary contribution 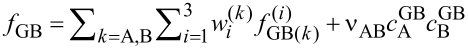, with respect to the variables 
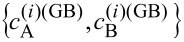
 subject to the two conservation laws; here 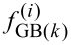 corresponds to 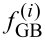 of Equation 1 for a binary alloy (component *k* in the matrix). The system separates into independent binary problems only at ν_AB_ = 0. (3) Strong segregations with competition for the same sites: Components A and B compete for identical sites at the GB. This situation is characteristic when atoms A and B have similar radii and both are either larger or smaller than the solvent atom M (consequently both components segregate either to sites of local expansion or to sites of local compression). For this case, we construct a more detailed generalization of the three-site model in the following.

The free-energy densities in the bulk *f*_b_ and at each of the sites on the GB are written in the regular-solution approximation, by analogy with Equation 1. For simplicity it is assumed that the interaction parameters ν_AA_, ν_BB_, ν_AB_ are the same everywhere:


[28]

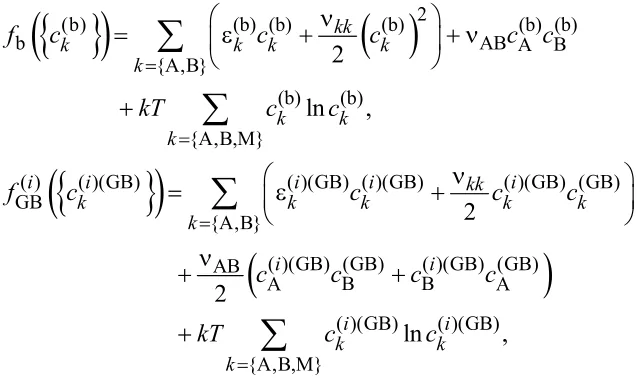



where




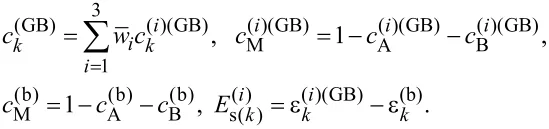




The free-energy density at the GB is, as before, the additive sum of the contributions of the individual sites:


[29]

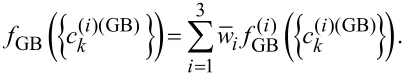



As a reasonable approximation for the weight coefficients, we adopt the average values 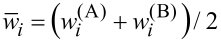, where 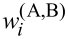 are the coefficients obtained for components A and B from Table 1. The physical meaning of this approximation is that, when both components segregate to the same lattice sites, the weight coefficients characterize the relative fractions of these sites and therefore should not depend on the atomic species.

The total free energy of the system is the sum of the bulk and grain-boundary contributions (see Equation 9); subject to the laws of conservation of matter, it is minimized with respect to the variables 
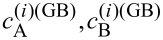
 in order to find their equilibrium values. As an example we consider the ternary alloy Ni–(Pd, Pt). The atomic radii of Pd (137 pm) and Pt (139 pm) are close to each other and substantially exceed the atomic radius of Ni (124 pm) [38]. This leads to strong competition of Pd and Pt for sites with large free volume at the GB. For the interaction parameters, we use the following values: ν_PdPd_ ≈ −20 kJ/mol [36], ν_PtPt_ ≈ +80 kJ/mol [39], and ν_PdPt_ ≈ +120 kJ/mol [40].

**Figure 8:**
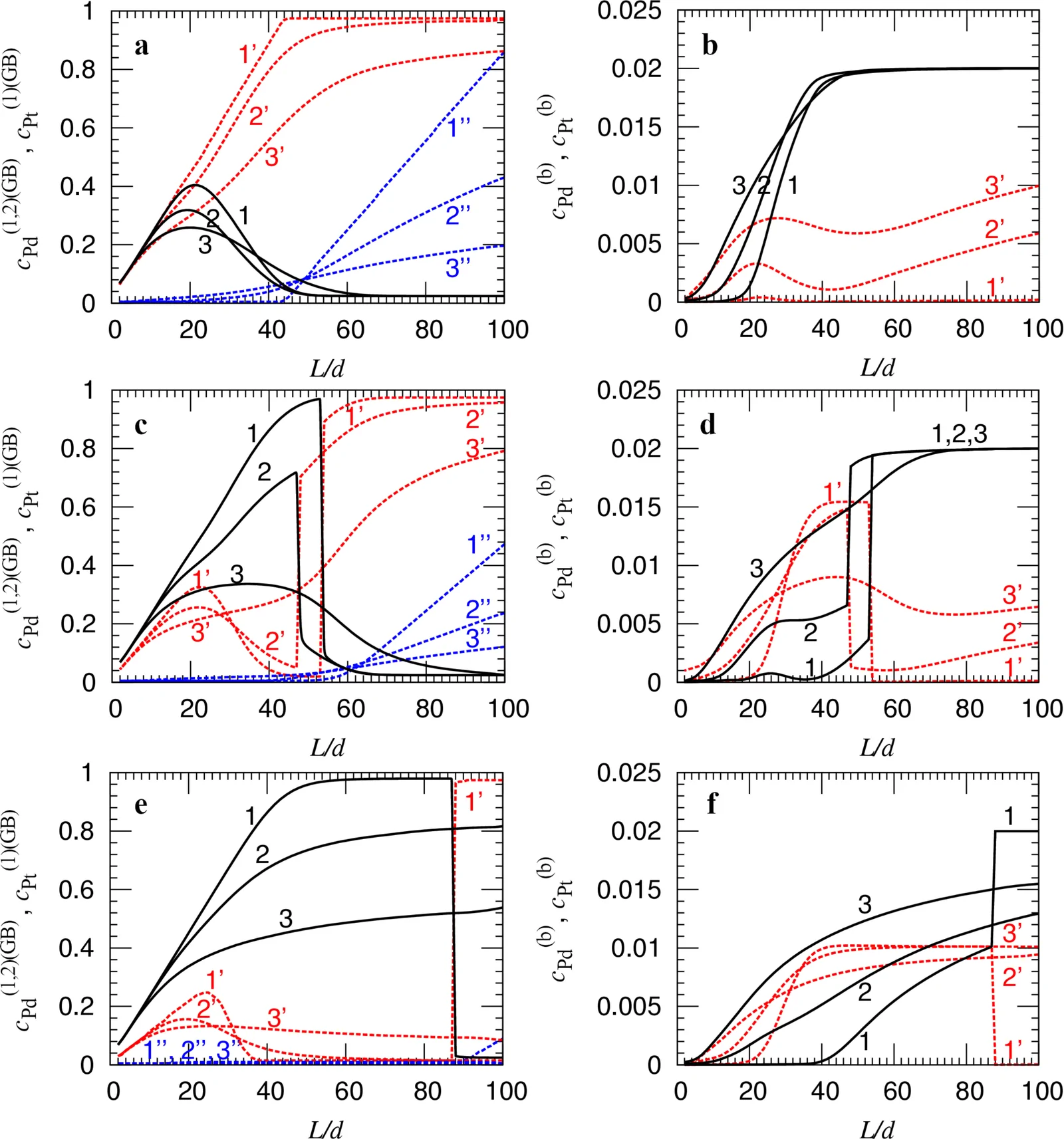
(a, c, e) Concentrations at the grain boundary 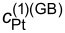 (curves 1–3), 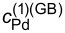 (curves 1′–3′) and 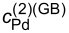 (curves 1″–3″) as functions of grain size at *T* = 300 K (1, 1′, 1″), 600 K (2, 2′, 2″), 900 K (3, 3′, 3″); (b, d, f) corresponding bulk concentrations 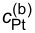 (curves 1–3) and 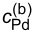 (curves 1′–3′). Average concentration 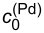 = 0.020 (a, b); 0.015 (c, d) 0.01 (e, f). In all cases, 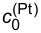 = 0.020.

Figure 8 shows the calculated equilibrium concentrations of Pd at sites 1 and 2 and of Pt at site 1 at the grain boundary (Figure 8a,c,e) and the corresponding bulk concentrations (Figure 8b,d,f) at three temperatures (*T* = 300, 600, and 900 K). The concentrations of Pd at site 3 and of Pt at sites 2 and 3 are negligibly small in all cases and are not presented. Figure 8a,b shows that the dependence 
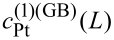
 is non-monotonic, while the dependence 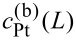 has a pronounced threshold character (up to a certain critical grain size, the Pt component remains “locked” in the bulk, where its concentration stays equal to the nominal average 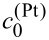). The critical size is associated with the exhaustion of Pd in the bulk: Upon further decrease of *L*, the concentration 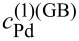 begins to fall and Pt atoms acquire the possibility of occupying the liberated sites at the GB. The qualitative effect of non-monotonic behavior of the concentration of one of the components at the GB as a function of grain size is not specific to the three-position model and is not exclusively related to the account of impurity interactions. Earlier, in [14], it was shown that this effect is observed even in an ideal ternary solid solution within the single-position model. A decrease of the average concentration 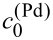 facilitates the segregation of Pt at the GB, and the observed picture becomes more complicated (Figure 8c–f). Below a certain temperature, upon reaching a critical grain size the model exhibits a discontinuous jump from one solution branch to another: 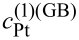 changes from values close to zero to almost unity (Figure 8c, curve 1), while 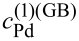 changes from values close to unity to almost zero (curve 1′). In an intermediate interval of grain sizes, the Pd component turns out to be “locked” in the bulk, where its concentration remains equal to 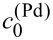 (Figure 8d, curve 1′). Upon further decrease of the grain size, 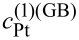 falls because of the exhaustion of Pt in the bulk, and the liberated sites at the GB are again filled by Pd atoms. Thus both concentrations 
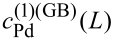
 and 
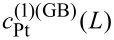
 display non-monotonic behavior (Figure 8c).

A further decrease of 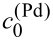 leads to the situation that, in the range of temperatures and grain sizes considered, Pt predominantly segregates, while Pd remains “locked” in the bulk until a critical size is reached (Figure 8e,f). Thus, depending on the ratio of the average concentrations 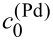 and 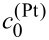, the components Pd and Pt largely exchange their roles.

Thus, the three-position model generalized to the case of competition of components for the same sites demonstrates new size effects in ternary alloys, that is, non-monotonic dependences of the concentrations on grain size, threshold (“locking”) phenomena, and discontinuous redistributions of the components between the bulk and the boundary. These features substantially enrich the picture of grain-boundary segregations in comparison with binary systems and single-site approximations.

### Suppression of decomposition in nanocrystalline alloys. Generalized phase diagram

In all the cases considered above, it was assumed that, upon reaching equilibrium, the impurity concentration in the bulk does not exceed the equilibrium solubility limit at the given temperature, 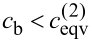, so that after the formation of grain-boundary segregations, the nucleation of precipitates in the grain interiors is impossible. In addition, it was assumed that the concentrations 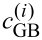 depend only on the type of site at the GB, while all sites of a given type are filled uniformly (i.e., two-dimensional decomposition directly at the GB does not occur). It was also shown that the equilibrium bulk concentration *c*_b_ increases with increasing grain size. Consequently, situations are possible in which grain refinement stabilizes the homogeneous state in the bulk through the formation of GBSs. This effect was previously investigated in [15] for a binary alloy, and a generalized phase diagram for a nanocrystalline alloy was constructed in which GBSs were described by the McLean–Fowler model taking into account the finite grain size. Below, a more consistent description of the effect is proposed that accounts for the interfacial energy of the precipitates and the specifics of the three-position model.

We seek the grain size *L*_dis_ at which, under conditions of thermodynamic equilibrium, the presence of a precipitate becomes energetically unfavorable. To this end, we equate the free energy of a sample containing a precipitate (*f*_dec_) to the free energy of a sample without a precipitate (*f*_ss_). In the presence of a precipitate, the equilibrium bulk concentration corresponds to the equilibrium solubility limit 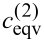, while, in the absence of a precipitate, it is determined by the equilibrium of the bulk with the GBSs and equals *c*_b_. Thus, the free energies *f*_ss_ and *f*_dec_ can be written as


[30]

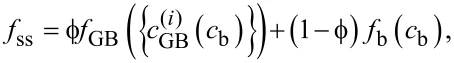




[31]

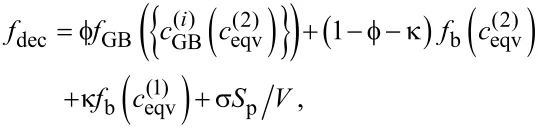



where κ is the volume fraction of the sample occupied by the precipitate, σ is the interfacial energy of the precipitate, *S*_p_ and *V* are the surface area of the precipitate and the sample volume, respectively, and 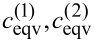 are the concentrations corresponding to the boundaries of the two-phase region (equilibrium concentration in the precipitate and in the matrix), respectively. If both the sample and the precipitate are spherical, then


[32] $$\frac{S_{\text{p}}}{V}=\frac{3R_{\text{p}}^{2}}{L^{3}}=\frac{3\kappa^{2/3}}{L},$$

where *R*_p_ is the radius of the precipitate.

In the absence of a precipitate, the equilibrium concentrations 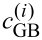, *c*_b_, are determined, as before, by minimization of the free energy of the sample (Equation 30) using the regular-solution expressions (Equation 1) and the conservation law (Equation 6). In the presence of a precipitate, the unknowns 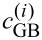
and *R*_p_ are found at fixed values of the concentrations in the precipitate and in the matrix, 
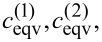
 while the conservation law takes the form:


[33] $$c_{\text{GB}}\phi +c_{\text{eqv}}^{\left(2\right)}\left(1-\phi -\kappa \right)+c_{\text{eqv}}^{\left(1\right)}\kappa =c_{0}.$$

From the condition *f*_ss_ = *f*_dec_ at the obtained equilibrium values of 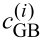 and *R*_p_, and at fixed (*T*, *c*_0_), one can find the critical size *L*_dis_(*T*, *c*_0_) below which the presence of a precipitate becomes energetically unfavorable. In addition, at fixed grain size *L* and average concentration *c*_0_, the dissolution of a precipitate occurs upon heating to a certain temperature that, owing to the presence of GBSs, differs from the temperature predicted by the binodal Equation 2. This makes it possible to construct a generalized phase diagram of a nanocrystalline alloy.

**Figure 9:**
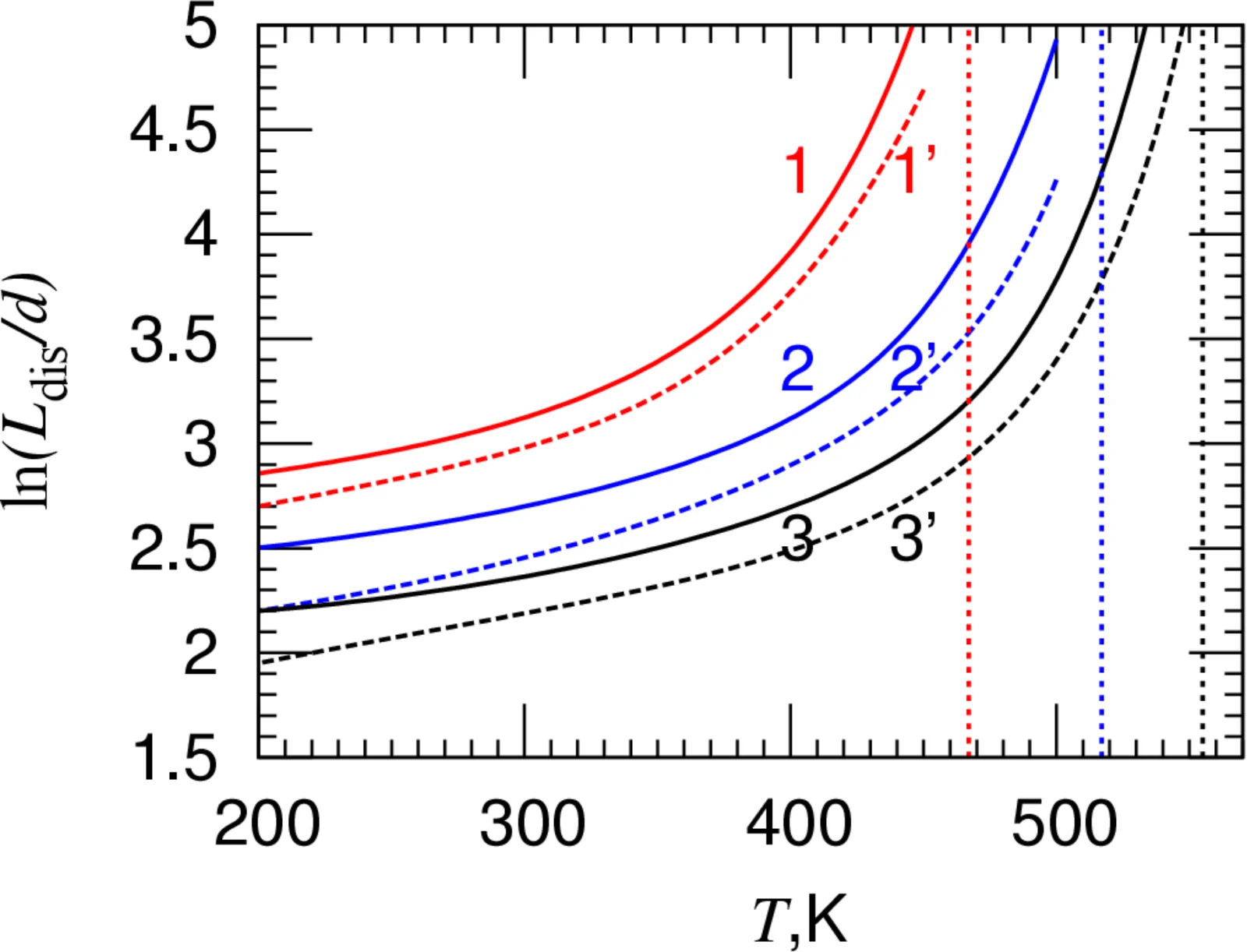
Temperature dependence of the critical size *L*_dis_ at which dissolution of precipitates occurs in the Ni–Cu alloy for copper concentrations *c*_0_ = 0.10 (1, 1′), 0.15 (2, 2′), 0.20 (3, 3′). Interfacial energy of the precipitate σ = 40 mJ/m^2^ (curves 1–3), 0 (curves 1′–3′). Vertical lines correspond to the binodal temperature at the respective concentrations.

The calculations were performed for the Ni–Cu alloy, which possesses a wide two-phase region at sufficiently low temperatures. For the Cu–Cu interaction energy in the Ni matrix the estimate ν = −21.3 kJ/mol is used, obtained from the experimentally known position of the top of the two-phase dome (≈630 K) and the relation *kT* = −ν_b_/4. For the interfacial energy of the precipitates, an approximate value σ = 40 mJ/m^2^ is adopted, by analogy with the known energies of coherent precipitate interfaces in the alloys Ni–Co and Ni–Al [41–42]. Figure 9 shows the temperature dependence of the critical size *L*_dis_(*T*) for different alloy compositions, accounting for (curves 1–3) and not accounting for (curves 1′–3′) the interfacial energy of the precipitates. The critical size decreases monotonically both with increasing average Cu concentration and with decreasing temperature. At the onset temperature of decomposition (the binodal condition, see Equation 2), it tends to infinity; upon undercooling of ≈50 K relative to this temperature, it amounts to ≈100 nm. Taking the interfacial energy of the precipitates into account proves to be significant and can lead to an approximately twofold increase of *L*_dis_.

**Figure 10:**
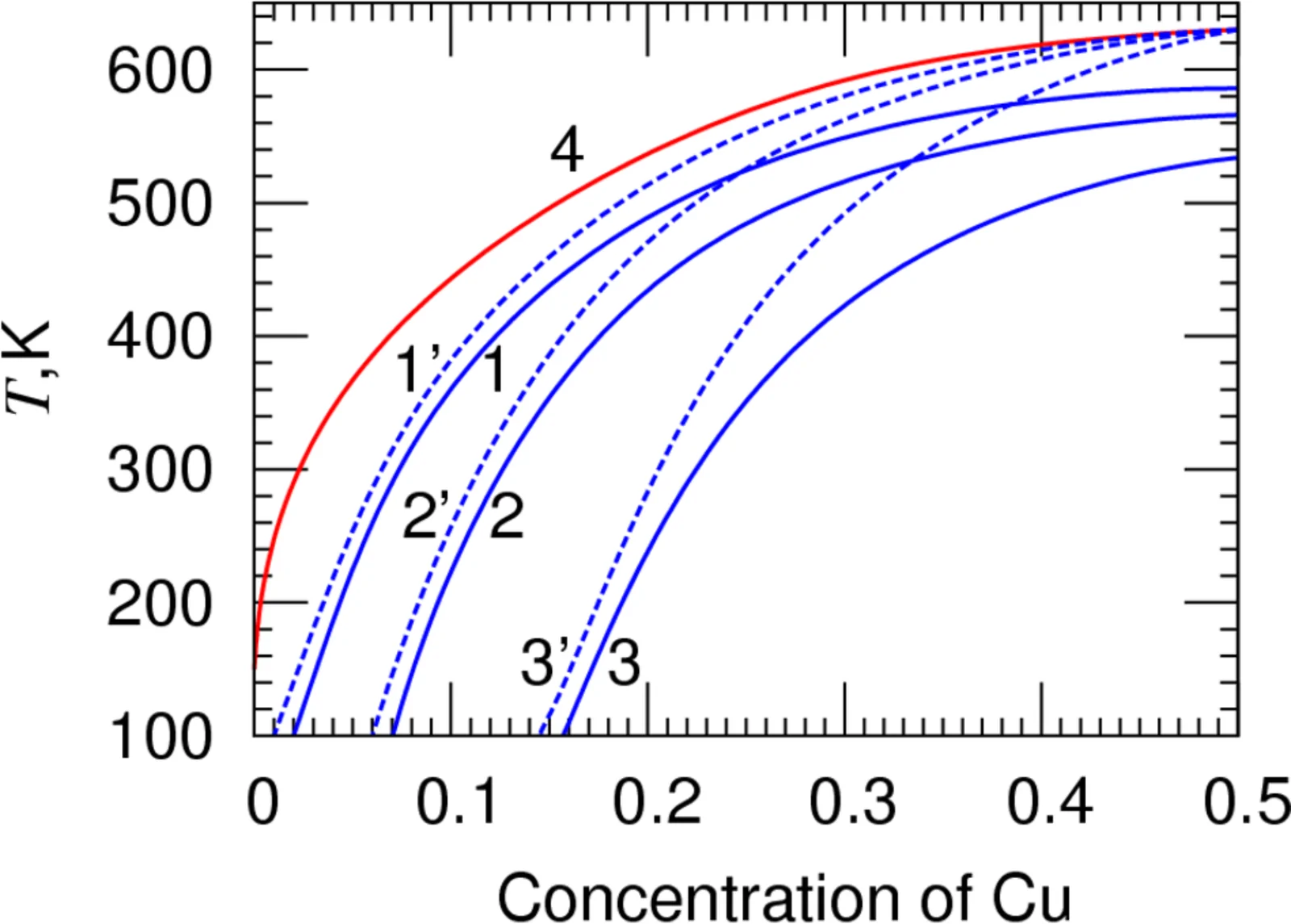
Generalized phase diagram of the Ni–Cu alloy (boundary of the two-phase region) at *L*/*d* = 40 (1, 1′), 20 (2, 2′), 10 (3, 3′). Interfacial energy of the precipitates σ = 40 mJ/m^2^ (curves 1–3), 0 (curves 1′–3′). Curve 4 is the binodal in the absence of grain-boundary segregations.

Figure 10 presents the generalized phase diagram of the Ni–Cu alloy in the nanocrystalline state. It can be seen that the presence of GBs stabilizes the homogeneous state in the bulk: The onset temperature of decomposition is lowered in fine grains (compare curves 1–3 for different grain sizes with the binodal 4 in the absence of GBs). Moreover, in sufficiently fine grains, decomposition is suppressed even in the limit *T* → 0 (curve 3); this effect was noted earlier in [15] within a simple model of GB segregations. Taking the interfacial energy of the precipitates into account noticeably expands the stability region, especially at small grain size and elevated concentrations (curves 1′–3′).

Thus the three-position model combined with accounting for the interfacial energy of precipitates shows that refinement of the grain structure can effectively suppress bulk decomposition through redistribution of the impurity to the boundaries. The generalized phase diagram of the nanocrystalline Ni–Cu alloy clearly demonstrates the expansion of the solid-solution stability region with decreasing grain size.

## Discussion

The proposed three-position model represents a natural generalization of the classical McLean and Fowler isotherms to the case of energetically inequivalent sites at the grain boundary while taking into account the finite grain size. The use of three discrete types of sites (with negative, near-zero, and positive segregation energy), whose parameters are matched to the first moments of the skew-normal spectrum of *E*_s_, makes it possible to retain analytical and computational transparency while simultaneously reproducing the key qualitative features of the continuous spectral approach. In particular, the model naturally describes the saturation of the average boundary concentration by the value *w*_1_ + *w*_2_ < 1 in the low-temperature limit, the successive filling/emptying of sites of different types with changing grain size, the non-monotonic dependences of the concentrations on *L*, and the effects of component competition in ternary systems.

The present description continues the spectrum-of-binding-energies approach introduced by White and Coghlan [19] and applied by White and Stein [20] to measurements of sulfur at grain boundaries in Ni_3_Al. In that work a single binding energy was replaced by a spectrum of site energies, arising from the site-to-site variation of the solute environment and estimated from the elastic interaction with tilt boundaries. The measured coverage is then an average over the spectrum, which accounts for a grain-boundary concentration that falls with temperature more slowly than a single-energy isotherm and for the corresponding increase of the effective binding energy with temperature. This early construction was implemented with an assumed Gaussian distribution of binding energies fitted to experiment. In contrast, in our model, the spectrum is taken from atomistic and machine-learning calculations and is represented by three site classes matched to the mean, variance, and asymmetry of the skew-normal distribution [27,29]; the analysis is extended to a finite grain size, to solute–solute interactions in the Fowler approximation, and to the competition of solutes in a ternary alloy.

A substantial advantage of the approach is the possibility of explicitly introducing the critical sizes 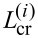 (purification of the grain interior, see, for example, Figure 5f) and 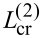 (maximum of the integral degree of demixing), as well as the size *L*_dis_ below which the dissolution of precipitates becomes thermodynamically favorable. These sizes turn out to depend not only on temperature but also on the average alloy composition; in ternary systems they additionally acquire a threshold and discontinuous character because of competition for the same sites. The generalized phase diagram of Ni–Cu demonstrates that the presence of grain boundaries with a discrete spectrum of segregation energies stabilizes the homogeneous solid solution down to *T* = 0 K at sufficiently small *L*/*d*; accounting for the interfacial energy of the precipitates noticeably expands the stability region.

Calculations performed for the systems Ni–Pd and Ni–(Pd, Pt) show that even moderate interaction energies substantially modify the curves 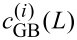 and *c*_b_(*L*) in the intermediate temperature range, whereas, in the low-temperature limit, the behavior is determined predominantly by the geometry of the available sites. This underlines the necessity of jointly accounting for the spectrum of *E*_s_ and the interaction parameters when analyzing real systems.

The model rests on a number of simplifications that should be kept in mind in quantitative estimates. First, it is assumed that all grain boundaries of a given grain are identical, whereas a real spectrum of *E*_s_ is constructed over the entire diversity of boundaries in a polycrystal. This leads to a dispersion of critical sizes that is ignored. Second, in the sections devoted to segregations in the region of solid-solution stability, the possibility of two-dimensional decomposition directly at the boundary is not considered. The obtained solutions retain the meaning of grain-boundary segregations, even when the average boundary concentration formally falls into the two-phase region of the bulk diagram, because all sites of a given type are regarded as uniformly occupied. Third, the approximation 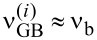 is adopted; a more accurate determination of the distribution of interaction energies at the boundary requires resource-intensive atomistic calculations that have so far been performed only for individual systems. Finally, at low temperatures, many of the predicted effects may be kinetically inaccessible because of extremely small diffusion coefficients (especially in the bulk). Nevertheless, the qualitative features, which can be observed even at intermediate temperatures, that is, non-monotonicity, “locking” of a component in the bulk, and intermediate segregation compositions, remain important guidelines for the interpretation of experiments and the planning of thermal treatments of nanocrystalline alloys. It should be noted that the low-temperature states of many alloys are reached under conditions of severe plastic deformation [43], which can raise diffusion coefficients by many orders of magnitude through the generation of a large number of non-equilibrium point defects. In particular, in the alloy Ni_41_Pd_59_, after ball-milling of the powder at *T* ≈ 400 K, an anomalous shift of the Curie temperature was observed, indicating substantial demixing of the alloy [44].

## Conclusion

A three-position thermodynamic model of grain-boundary segregations in binary and ternary alloys has been developed that explicitly takes into account the finite grain size and the energetic inequivalence of sites at the boundary. The model reproduces the principal moments of the skew-normal spectra of segregation energies and permits both an analytical treatment of dilute solutions and a numerical minimization of the free energy in concentrated systems. It has been shown that (1) with decreasing grain size the impurity is successively exhausted first in the bulk and then in the sites with segregation energies of smaller absolute value; (2) the average boundary concentration in the low-temperature limit tends to *w*_1_ + *w*_2_ < 1 rather than to unity; (3) the critical sizes 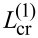 and 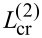 depend on the average composition and increase as the latter decreases; (4) in ternary alloys with competition for the same sites, non-monotonic dependences of the concentrations on *L* and “locking” effects of the components arise; and (5) the presence of grain boundaries with a discrete spectrum of segregation energies expands the solid-solution stability region, which is reflected in the generalized phase diagram of the nanocrystalline state.

The results obtained demonstrate that accounting for the energetic heterogeneity of grain boundaries substantially alters the picture of size effects of segregation and decomposition in comparison with classical single-position models and must be taken into consideration when analyzing the thermodynamic stability and properties of nanocrystalline alloys.

## Data Availability

All data that supports the findings of this study is available in the published article and/or the supporting information of this article.

## References

[1] Zhang X F, Fujita T, Pan D, Yu J S, Sakurai T, Chen M W (2010). Mater Sci Eng, A.

[2] Wong C Y, Grovenor C R M, Batson P E, Smith D A (1985). J Appl Phys.

[3] Fujita Y, Kitakizaki K, Masuda-Jindo K (1996). Solid State Phenom.

[4] Mandurah M M, Saraswat K C, Helms C R, Kamins T I (1980). J Appl Phys.

[5] Totten G E (2006). Steel Heat Treatment Handbook - 2 Volume Set.

[6] Lejček P, Šob M, Paidar V (2017). Prog Mater Sci.

[7] Beke D L, Cserháti C, Szabó I A (2004). J Appl Phys.

[8] Seah M P, Hondros E D (1973). Proc R Soc London, Ser A.

[9] Weissmüller J (1993). Nanostruct Mater.

[10] Ishida K (1996). J Alloys Compd.

[11] Slezov V V, Davydov L N, Rogozhkin V V (1995). Phys Solid State.

[12] Gornostyrev Y N, Razumov I K, Yermakov A Y (2004). J Mater Sci.

[13] Razumov I K, Gornostyrev Y N, Yermakov A Y (2007). J Alloys Compd.

[14] Razumov I K (2014). Russ J Phys Chem A.

[15] Razumov I K (2018). Russ J Phys Chem A.

[16] McLean D (1960). Grain Boundaries in Metals.

[17] Fowler R H, Guggenheim E A (1939). Statistical Thermodynamics.

[18] Christian J W (1975). The Theory of Transformations in Metals and Alloys, Part I.

[19] White C L, Coghlan W A (1977). Metall Trans A.

[20] White C L, Stein D F (1978). Metall Trans A.

[21] Chen S P, Voter A F, Srolovitz D J (1986). Scr Metall.

[22] Hashimoto M, Ishida Y, Wakayama S, Yamamoto R, Doyama M, Fujiwara T (1984). Acta Metall.

[23] Popov V V (2012). Phys Met Metallogr.

[24] Gaertner D, Wilde G, Divinski S V (2017). Acta Mater.

[25] Razumovskiy V I, Divinski S V, Romaner L (2018). Acta Mater.

[26] Wagih M, Schuh C A (2019). Acta Mater.

[27] Wagih M, Larsen P M, Schuh C A (2020). Nat Commun.

[28] Wagih M, Schuh C A (2021). Acta Mater.

[29] Tuchinda N, Schuh C A (2022). Acta Mater.

[30] Matson T P, Tuchinda N, Schuh C A (2026). Acta Mater.

[31] Azzalini A, Capitanio A (2013). The Skew-Normal and Related Families.

[32] Gupta K P (2009). J Phase Equilib Diffus.

[33] Azzalini A (2005). Scand J Stat.

[34] Wagih M, Schuh C A (2020). Acta Mater.

[35] Matson T P, Schuh C A (2021). Nanomaterials.

[36] Manzoor A, Pandey S, Chakraborty D, Phillpot S R, Aidhy D S (2018). npj Comput Mater.

[37] Guttmann M (1975). Surf Sci.

[38] Pauling L (1960). The Nature of the Chemical Bond.

[39] Botha L M, Santos-Carballal D, Terranova U, Quesne M G, Ungerer M J, van Sittert C G C E, de Leeuw N H (2019). RSC Adv.

[40] Chen S, Cotler J, Huang H-Y, Li J (2023). Nat Commun.

[41] Dasari S, Jagetia A, Chang Y-J, Soni V, Gwalani B, Gorsse S, Yeh A-C, Banerjee R (2020). J Alloys Compd.

[42] Mishin Y (2014). Modell Simul Mater Sci Eng.

[43] Razumov I K, Yermakov A Y, Gornostyrev Y N, Straumal B B (2020). Phys-Usp.

[44] Yermakov A Y, Gapontzev V L, Kondratyev V V, Gornostyrev Y N, Uimin M A, Korobeinikov A Y (2000). Mater Sci Forum.

